# A Rare Case of Idiopathic Capillary Leak Syndrome

**DOI:** 10.7759/cureus.14370

**Published:** 2021-04-08

**Authors:** Suman Rao, Omar Nasser, Akhila Sunkara, Rishi Singhal, Dragos Manta

**Affiliations:** 1 Internal Medicine, State University of New York Upstate Medical University, Syracuse, USA; 2 Critical Care, State University of New York Upstate Medical University, Syracuse, USA

**Keywords:** idiopathic capillary leak syndrome

## Abstract

Idiopathic capillary leak syndrome (ICLS) occurs as a result of vascular membrane instability, which results in the leakage of several proteins from the vascular compartment to the interstitial spaces. It is an extremely rare disorder, with around 260 cases documented thus far. We present a case of a 35-year-old male with a past medical history of asthma and gastroesophageal reflux disease who initially presented to our hospital for the treatment of chronic demyelinating inflammatory neuropathy requiring plasmapheresis and steroid therapy. After removal of his vascular catheter, he experienced sudden onset of dyspnea, hypotension, and respiratory distress. His lab work showed a hemoglobin of 21.3 g/dL and a hematocrit of 62.6%. Protein electrophoresis showed a mildly decreased albumin at 3.28 g/dL. These findings were consistent with ICLS. He required management with colloids and systemic steroids. The difficult diagnosis of ICLS is due to its overlap with several medical emergencies, such as sepsis and anaphylaxis. Further studies are required to study the role of steroids and colloids in the management of ICLS.

## Introduction

Idiopathic capillary leak syndrome (ICLS) is an extremely rare disorder, with around 260 cases documented worldwide [1]. As its name implies, at the cornerstone of the pathogenesis of this disease is the instability of the vascular membrane that results in the leakage of several proteins from the vascular compartment to the interstitial spaces [2]. There are not many studies in the literature that aim to explain the pathogenesis of disease. The management of ICLS is largely extrapolated from the management of distributive shock.

## Case presentation

We present a case of a 35-year-old male with a past medical history of asthma and gastroesophageal reflux disease who initially presented to the neurological ICU at our hospital for further management of neuropathic pain and focal neurological deficits.

On initial presentation, our patient complained of a 10-week history of muscle soreness, stabbing gluteal pain, paresthesia of the upper and lower limbs bilaterally, left-sided facial weakness, and lower extremity weakness. Lab work was significant for a hemoglobin of 13.0 g/dL, hematocrit of 39.1%, and a creatinine of 0.93 mg/dL. MRI of the cervical and thoracic spine showed no abnormalities. Cerebrospinal fluid analysis showed normal cytology, normal protein content, and absence of oligoclonal bands. Outpatient electromyography showed demyelinating neuropathy. He was diagnosed with chronic demyelinating inflammatory neuropathy. After placement of a vascular catheter, he was managed with five sessions of plasmapheresis and a five-day course of 500 mg IV methylprednisolone. He showed a drastic improvement in his symptoms.

On the day of discharge, his vascular catheter was removed. Shortly thereafter, he began to experience shortness of breath and dizziness. His systolic blood pressure dropped to 61 mmHg. He desaturated to an oxygen saturation of 73%. Physical examination at this time showed a diaphoretic man in cardiopulmonary distress, with rhonchi and adventitious breath sounds. He required 15 L of oxygen via a nasal cannula to maintain a saturation of 94%. He was given a bolus of 2 L of normal saline, after which his blood pressure was recorded to be 94/59 mmHg with a mean arterial pressure (MAP) of 75 mmHg.

**Figure 1:**
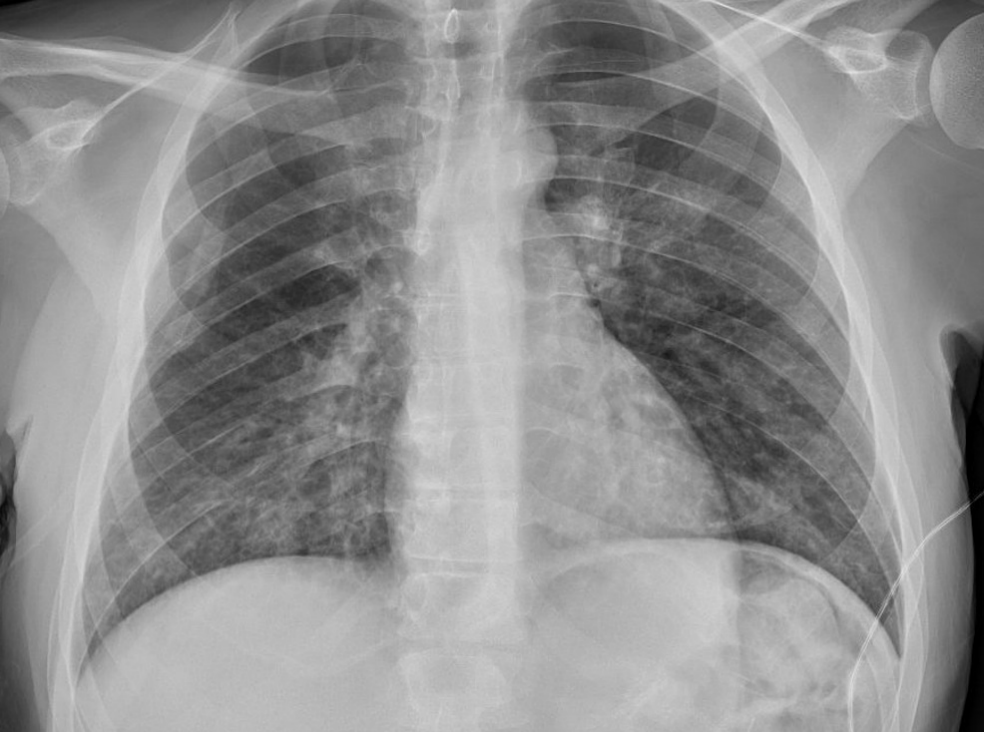
Chest radiograph after initial episode of desaturation, which did not show any abnormalities.

**Figure 2:**
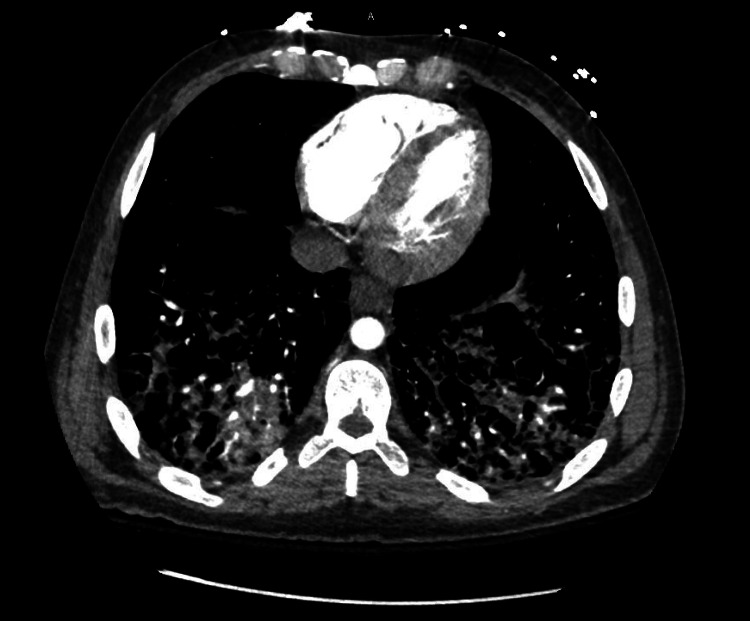
CT of the thorax after initial episode of desaturation, which was negative for any perfusion defects but did show ground-glass opacities.

A chest X-ray was performed, which did not show any acute abnormalities. No pneumothorax was noted (Figure 1). A computed tomography angiography (CTA) was also performed, which was negative for pulmonary embolism but did show ground-glass opacities (Figure 2). He received 40 mg IV furosemide for presumed transfusion-related acute lung injury (TRALI) and was transferred to the medical ICU for further management of his acute symptoms.

Further lab work showed a hemoglobin of 21.3 g/dL and a hematocrit of 62.6%. Creatinine was noted to rise to 1.05 mg/dL. Arterial blood gas (ABG) showed pH of 7.35, pCO_2_ of 44 mmHg, and pO_2_ of 60 mmHg. Immunoglobulin (Ig) assay revealed a decreased IgG at 270 mg/dL, with normal IgA and IgM levels. Serum protein electrophoresis showed an abnormal band in the beta region overlapping with normal beta globulins (estimated at 0.16 g/dL). Mild hypoalbuminemia (3.28 g/dL), decreased alpha 2 globulins (0.31 g/dL), and moderate hypogammaglobulinemia (0.28 g/dL) were also noted.

Given the hemoconcentration, hypoalbuminemia, and initial hypotension, our patient was diagnosed with ICLS. He was started on IV albumin 25% 25 g every six hours to help raise oncotic pressure. He required pressor support with phenylephrine and subsequently epinephrine to maintain a MAP of 65 mmHg. Management of blood pressure was focused on pressure support and not fluid resuscitation, as fluid would come back into the vessels during the fluid recruitment phase of ICLS. He also required continuous positive airway pressure (CPAP) at 80% fiO_2_ to maintain appropriate oxygen saturation. We continued with IV methylprednisolone 40 mg daily.

The next day, our patient was able to be weaned down to 6 L/min of oxygen via nasal cannula. He no longer had pressor requirements. His hemoglobin and hematocrit came down to normal at 15.5 g/dL and 45%, respectively. Creatinine trended down to 0.83 mg/dL. Our patient was switched over to 60 mg prednisone daily.

He was transferred to the medical floor and was able to maintain appropriate oxygen saturation on room air. He was discharged with a prednisone taper in order to avoid any complications of adrenal insufficiency and was given a referral to the immunology service.

## Discussion

The etiology of ICLS remains unexplained. With regard to epidemiology, it appears that the disease does not discriminate between age and gender groups [3].

There are several theories that aim to explain the pathogenesis of ICLS. Vascular endothelial growth factor (VEGF), angiopoietin-2, IL-2, leukotrienes, and endothelial cell apoptosis have been thought to be implicated in instigating the disease process [4-7]. It can be inferred that the capillary leak is largely drawn by the release of the cytokines.

At first, our suspicion was the presence of an air embolus that could potentially lead to a release of cytokines after damage to the capillary alveolar interface. However, an air embolus alone would not explain the systemic and diffuse nature of the presentation in our patient, including hypotension and acute kidney injury.

ICLS manifests itself in three defined phases. The first phase, or the prodromal phase, consists of nonspecific symptoms such as fatigue, bilateral lower extremity edema, or myalgia [8]. The fluid extravasation phase consists of the triad of hypotension, hemoconcentration, and hypoalbuminemia. Nearly all patients will present with hypotension, as there will be a migration of vascular fluid into the interstitial space. Loss of oncotic pressure further contributes to the hypotension. Hemoconcentration occurs as a result of loss of fluid, with red blood cell products largely intact within the vascular space. Hypoalbuminemia occurs from increased permeability of the capillary surface [9]. The last phase is the fluid recruitment phase, which occurs as a result of decreased circulating cytokines, and the flow of fluid back into the vascular space.

The diagnosis of ICLS becomes difficult given its overlap with other conditions such as sepsis, anaphylaxis, and angioedema. What further complicates this diagnosis is that it is a diagnosis of exclusion. Diagnostic criteria for this syndrome are the three aforementioned clinical manifestations: presence of hypotension, hemoconcentration, and hypoalbuminemia.

The management of this syndrome is largely derived from the management recommendations from distributive and septic shock. First and foremost, hemodynamic stabilization is key. As in our patient, increasing oxygen requirements are common and could even progress to requiring intubation. In the fluid extravasation phase, fluid repletion is recommended as well as pressure support to ensure adequate perfusion to all organs of the body. Close monitoring is required for these patients as they can abruptly move on to the fluid recovery phase, which can be seen with decreased pressor support. At that time, it is essential to decrease maintenance fluids so as not to induce a fluid overloaded state. There are studies that have tried to assess the use of intravenous immunoglobulin (IVIG) for treatment; however, no improved survival was seen [10].

For the management of our patient, we found that systemic steroids in addition to adequate fluid resuscitation (with a combination of colloids and crystalloids) was required. Further studies should focus on the efficacy of steroids and colloids in the management of ICLS.

## Conclusions

ICLS is a rare occurrence, with less than 300 cases documented thus far. The difficult diagnosis of this syndrome is not only due to it being a diagnosis of exclusion but also because of its overlap with several medical emergencies. Further studies are required to assess the effectiveness of steroids and colloids in the management of ICLS in addition to adequate management of systemic perfusion.
